# Area postrema syndrome in patients with autoimmune glial fibrillary acidic protein astrocytopathy

**DOI:** 10.3389/fneur.2025.1538602

**Published:** 2025-01-30

**Authors:** Qingchen Li, Junfang Teng

**Affiliations:** Department of Neurology, The First Affiliated Hospital of Zhengzhou University, Zhengzhou, China

**Keywords:** glial fibrillary acidic protein, area postrema syndrome, encephalitis, magnetic resonance imaging, aquaporin-4

## Abstract

**Objective:**

Area postrema syndrome (APS) is a relatively rare symptom of autoimmune glial fibrillary acidic protein astrocytopathy (A-GFAP-A). This study aimed to report the APS in GFAP-immunoglobulin G (GFAP-IgG) positive patients.

**Methods:**

We retrospectively analyzed the clinical data of APS in GFAP-IgG positive patients and reviewed relevant literature. Moreover, we compared these data with APS patients in aquaporin-4-IgG-positive neuromyelitis optica spectrum disorders (AQP4-IgG+ NMOSD).

**Results:**

7 of 75 (9.3%) GFAP-IgG positive patients experienced APS, including 4 females and 3 males. The median age of onset was 42 years (range, 12–71 years). All patients presented with APS as their initial manifestation. Nausea and vomiting were observed in all 7 patients, while hiccups occurred in 5 patients. The median duration of APS episodes was 12 days (range, 6–40 days). None of the patients experienced isolated APS episodes during their illness. AQP4-IgG was positive in 2 patients. 5 patients had dorsal medulla oblongata lesions, while 3 patients showed an “inverted V” sign on axial images. In addition, 5 patients presented with longitudinally extensive linear or patchy lesions in cervical spinal cord extending to area postrema on sagittal images. All APS attacks completely disappeared after immunotherapy. Compared with the APS in AQP4 + NMOSD, APS in A-GFAP-A had a lower proportion of females (33.3% vs. 80%, *p* = 0.003), more hiccups (81% vs. 50%, *p* = 0.037), more leptomeningeal enhancement (61.9% vs. 5%, *p* = 0.000), higher CSF white blood cell count (median 120 vs. 10 cells/mm^3^, *p* = 0.000) and protein (median 0.949 vs. 0.407 g/L, *p* = 0.000). Furthermore, fewer patients with A-GFAP-A received long-term immunotherapy (19% vs. 65%, *p* = 0.003).

**Conclusion:**

APS often occurs as an initial manifestation of A-GFAP-A. MRI examination and antibody testing should be performed in suspected patients to avoid misdiagnosis.

## 1 Introduction

Autoimmune glial fibrillary acidic protein astrocytopathy (A-GFAP-A) is a severe autoimmune inflammatory disorder of the central nervous system (CNS) first described by Lennon et al. (1, 2). This disorder primarily affects the brain, meninges, spinal cord, and optic nerve, leading to various clinical syndromes such as encephalitis, meningitis, myelitis, and visual impairment. GFAP immunoglobulin G (IgG) has been considered a highly specific biomarker in the diagnosis of the disease (1–4).

The area postrema syndrome (APS), which refers to otherwise unexplained intractable nausea, vomiting, and hiccups (INVH) lasting longer than 48 h, is a characteristic presentation of neuromyelitis optica spectrum disorders (NMOSD) and was included in the international consensus diagnostic criteria for NMOSD in 2015 (5, 6). In recent years, some literature reports have found that APS appears not only in demyelinating diseases but also in infections, tumors, poisonings, and metabolic diseases (7–9). In 2018, Ciron et al. first reported a case of a female patient who developed APS associated with anti-GFAP encephalomyelitis, expanding the clinical manifestations of this disorder (10). However, in contrast to NMOSD, APS is not frequently observed as a symptom of A-GFAP-A. Most of the previous studies were case reports and no studies focused on comparing the differences between APS in A-GFAP-A and in NMOSD (11–19). Herein, we reported a case series of GFAP-IgG positive patients who developed APS and reviewed relevant literature. Furthermore, we compared the clinical data of APS in A-GFAP-A and NMOSD to enhance our understanding of this phenomenon.

## 2 Patients and methods

### 2.1 Patients

The study followed the ethical guidelines and was approved by the Ethics Committee of The First Affiliated Hospital of Zhengzhou University (2024-KY-2298).

In this retrospective observational study, we identified 75 patients who tested positive for GFAP-IgG and were admitted to The First Affiliated Hospital of Zhengzhou University from February 2020 to May 2024. They all met the inclusion criteria: (1) CSF or/and serum positivity for GFAP-IgG by tissue-based assays and cell-based assays, (2) one or more episodes of encephalitis, meningitis, myelitis, and optic neuritis, (3) clinical data available. Exclusion criteria: other disease, such as brain tumors, brain traumatic, Alzheimer’s disease, toxic and metabolic diseases of the CNS. In addition, aquaporin-4 (AQP4)-IgG and myelin oligodendrocyte glycoprotein (MOG)-IgG were detected by cell-based assays in all patients. APS was defined as the following criteria: (1) acute or subacute, single or combined, intractable nausea, vomiting, or hiccups, (2) lasting more than 48 h, (3) etiology was unknown (5, 6). Of the 75 GFAP-IgG positive patients, 7 developed APS throughout the course of the disease. In contrast, we selected NMOSD patients with APS as the control group. The inclusion criteria were as follows: (1) one or more episodes of APS during the course of the disease, (2) CSF or/and serum positivity for AQP4-IgG, (3) CSF or/and serum negativity for GFAP-IgG and MOG-IgG, (4) Clinical data available. Two experienced neurologists arrived at the diagnosis independently.

### 2.2 Data collection

We reviewed patients’ medical records, including demographics, acute clinical manifestations, magnetic resonance imaging (MRI) features, CSF analysis, treatment, and outcomes. Information on the duration of INVH, first visit department, accompanying other neurological symptoms, and interval from INVH to the appearance of other neurological symptoms were collected. The modified Rankin Scale (mRS) (20) was used to assess the disease severity and clinical outcomes at the last follow-up, and mRS<3 was considered a good outcome.

### 2.3 Neuroimaging

The patients underwent examinations using a 3.0 T MRI machine for head and spinal cord scans. MRI scans were evaluated for brain and spinal cord lesions using T1-weighted imaging (T1WI), T2-weighted imaging (T2WI), and fluid-attenuated inversion recovery (FLAIR) sequences in axial, coronal, and sagittal views. The imaging was assessed by two independent radiologists who reached a consistent conclusion.

### 2.4 Literature review

We searched PubMed through December 2024 for articles published in any language with the search string (“glial fibrillary acid protein” [MeSH Terms] OR “GFAP”) AND (“area postrema” [MeSH Terms] OR “AP” OR “APS” OR “nausea” OR “vomiting” OR “hiccups”). We also searched the references for related published articles. All obtained articles were reviewed to identify the cases of APS in A-GFAP-A.

### 2.5 Statistical analysis

Statistical analyses were conducted using SPSS version 26.0. Continuous variables were described by mean ± standard deviation (X ± *s*) or median (range). Categorical variables were described by counts and proportions. A Mann–Whitney *U* test or a t-test was used for continuous variables, and a chi-square test or Fisher’s exact test was used for categorical variables. *p* < 0.05 was considered statistically significant.

## 3 Results

### 3.1 Clinical characteristics of GFAP-IgG positive patients with APS

#### 3.1.1 Demographics and manifestations

7 of 75 (9.3%) GFAP-IgG positive patients experienced APS, including 4 females and 3 males. The median age at onset was 42 years (range, 12–71 years), with 1 patient being under the age of 18.1 patient (Patient 1) had esophageal cancer prior to the onset of neurological symptoms, whereas the other patients did not have any tumors. 2 patients were positive for AQP4-IgG, while none of patients were positive for MOG-IgG. 4 patients were tested for N-methyl-D-aspartate receptor (NMDAR)-IgG, and all were negative.

All patients presented with APS as their initial manifestation. Nausea and vomiting were observed in all 7 patients, while hiccups occurred in 5 patients. The median duration of APS episodes was 12 days (range, 6–40 days). 3 patients initially experienced delayed diagnosis or were misdiagnosed. They first visited the gastroenterology department and underwent extensive examinations such as gastroscopy, gastrointestinal contrast, and abdominal CT. However, the underlying etiology was not determined at that time. They received the correctly diagnosed after developing other neurological symptoms.

None of the patients experienced isolated APS episodes. The median interval from INVH occurrence to other neurological symptoms was 4 days (range, 0–120 days). The predominant clinical phenotypes included encephalomyelitis (3), meningoencephalomyelitis (2), meningoencephalitis (2), and optic neuritis (2). 2 patients who were AQP4-IgG positive also met the criterion of NMOSD. In addition to APS, Patient 5 had acute myelitis, acute brainstem syndrome and optic neuritis and Patient 6 had acute myelitis and acute brainstem syndrome. 6 patients experienced a single APS attack during the clinical course, whereas 1 patient encountered two APS attacks with an interval of 4 months. The clinical characteristics of these 7 patients are shown in Table 1.

**Table 1:** Clinical date of GFAP-IgG positive patients with APS.

No./Sex/Age (y)	Onset with APS	INVH	Duration of INVH (d)	Other symptoms	Interval from INVH to other symptoms (d)	MRI findings	AQP4-IgG serum/CSF	CSF WBC cells/mm^3^ protein, g/L	Immunotherapy	mRS max/last-follow-up
1/F/59	Yes	Vomiting, nausea	12	Fever, dizzy, tremor	0	Brain: medulla oblongata linear enhancement Spinal cord: cervical cord linear enhancement	−/−	WBC: 54 Protein: 0.837 OCBs: +	IVMP	3/1
2/M/71	Yes	Vomiting, nausea, hiccups	6	Fever, Headache, dizzy, psychiatric symptom consciousness disturbance, respiratory failure, neck stiffness	0	Brain: normality Spinal cord: C3	−/−	WBC: 68 Protein: 0.757 OCBs: +	IVMP	5/4
3/M/57	Yes	Vomiting, nausea, hiccups	40	Horner Syndrome, facial pain, cognitive dysfunction, paresthesia, hemiplegia, oculomotor dysfunction	50	Brain: frontal lobe, basal ganglia, lateral ventricle, corpus callosum, pons and medulla oblongata Spinal cord: normality	−/−	WBC: 70 Protein: 0.453 OCBs: -	IVMP	4/1
4/F/42	Yes	Vomiting, nausea, hiccups	30	Vision loss, papilloedema	120	Brain: medulla oblongata Spinal cord: C1-2 optic chiasm and optic tract	−/−	WBC: 6 Protein: 0.389 OCBs: +	IVMP, IVIG	3/1
5/F/12	Yes	Vomiting, nausea, hiccups	16	Fever, ear pain, facial paralysis, oculomotor dysfunction, paresthesia, vision loss	20	Brain: pons, medulla oblongata, lateral ventricle Spinal cord: C1-T2, T7, T10-11 binocular nerve	+/+	WBC: 31 Protein: 0.443 OCBs: +	IVMP, IVIG, MMF, RTX	4/2
6/F/34	Yes	Vomiting, nausea	11	Fever, dizzy, oculomotor dysfunction, ataxia	4	Brain: frontotemporal, medulla oblongata Spinal cord: T2-3, T12-L1	−/+	WBC: 87 Protein: 0.212 OCBs: −	IVMP, MMF	3/0
7/M/35	Yes	Vomiting, nausea, hiccups	10	Fever, headache, dizzy, neck stiffness, paralysis, paresthesia, consciousness disturbance, neck stiffness	0	Brain: leptomeningeal enhancement Spinal cord: C3-7, T7-12	−/−	WBC: 468 Protein: 0.831 OCBs: -	IVMP, IVIG	5/3

APS, the area postrema syndrome; AQP4, aquaporin-4; CSF, cerebrospinal fluid; F, female; GFAP, glial fibrillary acidic protein; IgG, immunoglobulin G; INVH, intractable nausea, vomiting, and hiccups; IVIG, intravenous immunoglobulins; IVMP, intravenous methylprednisolone; M, male; Max, maximum; MMF, mycophenolate mofetil; MRI, magnetic resonance imaging, mRS: modified Rankin Scale; OCBs, oligoclonal bands; RTX, rituximab; WBC, white blood cell.

#### 3.1.2 MRI imaging and CSF findings

**Figure 1:**
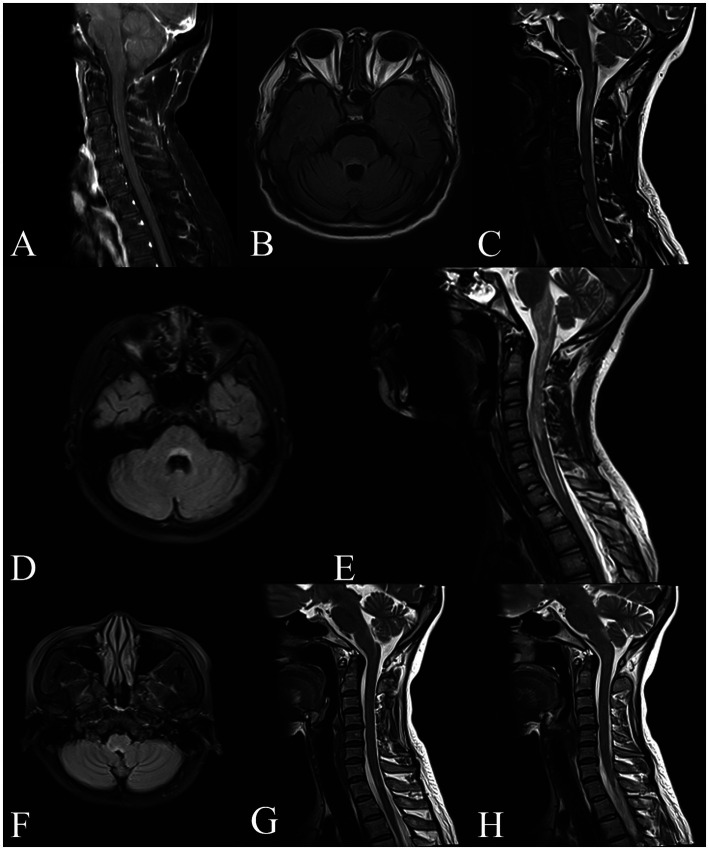
Brain and spinal cord MRI of GFAP-IgG positive patients with APS. Patient 1, **(A)** Sagittal T1-weighted MRI with gadolinium shows linear enhancement along the surface of the brainstem and in cervical spinal cord. Patient 3, **(B)** Axial T2-FLAIR imaging shows the lesion around the fourth ventricle. Patient 4, **(C)** Sagittal T2-weighted imaging shows a linear lesion in the medulla oblongata and upper cervical cord. Patient 5, **(D,E)** Axial T2-FLAIR imaging shows the “inverted V” lesion in the dorsal medulla oblongata involving the area postrema. Sagittal T2-weighted imaging shows a longitudinally extensive patchy lesion in the upper spinal cord extending to medulla oblongata. Patient 6, **(F,G,H)** Axial T2-FLAIR imaging shows the “inverted V” lesion in the dorsal medulla oblongata. Sagittal T2-weighted imaging shows the linear medullary lesion and the area postrema lesion.

Brain and spinal cord MRI scans were performed in all 7 patients. The median interval from INVH occurrence to MRI examination was 3 days (range, 1–120 days). 5 patients had T2WI/FLAIR hyperintense lesions in the dorsal medulla oblongata adjacent to the fourth ventricle, while 3 patients showed an “inverted V” sign on axial images. 5 patients presented with longitudinally extensive linear or patchy lesions in cervical spinal cord extending to area postrema on sagittal images. 4 patients had a linear medullary lesion or a linear medulla-spinal lesion. Gadolinium enhanced MRI was performed in all patents, and 6 patients had enhanced lesions. Of them, 2 patients had leptomeningeal enhancement and 1 patient (Patient 7) had periventricular linear enhancement. Aside from the medulla oblongata, there were abnormalities in the frontotemporal, basal ganglia, lateral ventricle, corpus callosum, pons, optic nerve, and thoracic spinal cord. The MRI characteristics of the 7 patients are shown in Table 1 and Figure 1.

CSF analysis revealed inflammation in all patients: Pleocytosis (>5 cells/mm^3^) was found in 7 patients, increased protein (>0.45 g/L) in 4 patients and oligoclonal bands were positive in 4 patients. Additionally, Epstein–Barr virus nucleic acid was detected in the CSF of 2 patients.

#### 3.1.3 Treatment and outcomes

In the acute stage, all patients received intravenous methylprednisolone (IVMP, 0.5–1 g/day for 5 days in 6 patients; 40 mg/day for 2 weeks in Patient 1) and subsequent oral methylprednisolone (48 mg/day), which was then tapered (decreased 4 mg/day every 1–2 weeks). 3 patients simultaneously received intravenous immunoglobulin (IVIG, 0.4 g/kg/d for 5 days). Additionally, 2 patients received long-term immunotherapy: one with mycophenolate mofetil, and the other with mycophenolate mofetil and rituximab. After treatments, all APS attacks completely disappeared. 4 patients experienced a relapse, with 1 patient having an APS episode (Patient 1, 4 months later).

### 3.2 Literature review

We identified 20 reported cases with APS in GFAP-IgG–positive patients by literature review, while 18 of them had available clinical data. All 18 patients were tested for AQP4-IgG, with 2 patients excluded who tested positive for AQP4-IgG. The APS in A-GFAP-A (21 cases including 5 cases in our cohort and 16 cases in literature review) compares to APS in AQP4 + NMOSD are shown in Table 2.

**Table 2:** Comparison of ASP in A-GFAP-A and AQP4 + NMOSD.

	Cases in this study (*n* = 5)	Cases in literature review (*n* = 16)	Total cases (*n* = 21)	AQP4 + NMOSD (*n* = 20)	*P-*vaule
Age at onset, median(range), y	57 (35, 71)	46 (21, 7)	46 (21, 76)	39 (16, 62)	0.103
Female, n(%)	2 (40)	5 (31.3)	7 (33.3)	16 (80)	0.003*
Isolated APS, n(%)	0	1 (6.3)	1 (4.8)	1 (5)	1.000
APS, n(%)					
Nausea	5 (100)	9 (56.3)	14 (66.7)	19 (95)	0.058
Vomiting	5 (100)	9 (56.3)	14 (66.7)	19 (95)	0.058
Hiccups	4 (80)	13 (81.3)	17 (81)	10 (50)	0.037*
Clinical manifestation, n(%)					
Other encephalopathy	5 (100)	15 (93.8)	20 (95.2)	15 (75)	0.164
Myelitis	3 (60)	12 (75)	15 (71.4)	15 (75)	0.796
Visual impairment	1 (20)	4 (25)	5 (23.8)	6 (30)	0.655
MRI lesion, n(%)					
Brain	4 (80)	14 (87.5)	18 (85.7)	17 (85)	1.000
Medulla oblongata or AP	3 (60)	7 (43.8)	10 (47.6)	15 (75)	0.072
Spinal cord	4 (80)	8 (50)	12 (57.1)	14 (70)	0.393
Cervical cord extending to AP	3 (60)	4 (25)	7 (33.3)	12 (60)	0.087
Leptomeningeal enhancement	2 (40)	11 (68.8)	13 (61.9)	1 (5)	0.000*
CSF examination					
WBC, [cells/mm^3^, median(range)]	68 (6, 468)	147 (16, 500)^a^	120 (6, 500)^a^	10 (2, 71)	0.000*
Protein, [g/L, median(range)]	0.757 (0.389,0.837)	1.19 (0.218, 4.063)^a^	0.949 (0.218, 4.063)^a^	0.407 (0.214, 1.039)	0.000*
Immunotherapy, n(%)					
IVMP	5 (100)	16 (100)	21 (100)	19 (95)	0.488
IVIG	2 (40)	3 (18.8)	5 (23.8)	7 (35)	0.413
Plasma exchange	0	0	0	1 (5)	0.488
Long-term immunotherapy	0	4 (25)	4 (19)	13 (65)	0.003*
Good outcome (mRS ≤ 3), n(%)	3 (60)	14 (87.5)	17 (81)	19 (95)	0.370
Relapse of APS, n(%)	1 (20)	1 (6.3)	2 (9.5)	6 (30)	0.208

^a^1 patient unknown; *P*-vaule, total cases compared with AQP4+ NMOSD.

* With statistical significance, A-GFAP-A, autoimmune glial fibrillary acidic protein; APS, the area postrema syndrome; AQP4, aquaporin-4; CSF, cerebrospinal fluid; IVIG, intravenous immunoglobulins; IVMP, intravenous methylprednisolone; MRI, magnetic resonance imaging; mRS, modified Rankin Scale; NMOSD, neuromyelitis optica spectrum disorders, WBC, white blood cell counts.

Compared with the patients with APS in AQP4 + NMOSD, patients with the APS in A-GFAP-A had a lower proportion of females (33.3% vs. 80%, *p* = 0.003), more hiccups (81% vs. 50%, *p* = 0.037), more leptomeningeal enhancement (69.1% vs. 5%, *p* = 0.000), higher CSF white blood cell count (median 120 vs. 10 cells/mm^3^, *p* = 0.000) and protein (median 0.949 vs. 0.407 g/L, *p* = 0.000), Furthermore, fewer patients with A-GFAP-A received long-term immunotherapy (19% vs. 65%, *p* = 0.003).

## 4 Discussion

GFAP is an intermediate filament protein primarily found in astrocytes within the CNS and is actively involved in various aspects of CNS physiology and pathology (21, 22). Autoantibodies that selectively target GFAP in astrocytes may trigger a persistent autoimmune attack, leading to a CNS autoimmune disease defined as A-GFAP-A (1, 2). The clinical manifestations of A-GFAP-A are diverse and non-specific, with symptoms that correlate with the location and size of the lesions. This disorder often presents with symptoms such as fever, headache, psychiatric symptoms, seizures, ataxia, meningeal symptoms, and myelopathic symptoms. In recent years, several studies have reported APS in A-GFAP-A, suggesting that APS is not rare in this disease (10–19). Deng et al. found that APS was present in 11% of A-GFAP-A patients, with 4% experiening APS as the initial symptom (11). Long et al. analyzed the data of 19 GFAP-IgG positive patients and found that 1 patient (5.3%) experienced intractable hiccups and nausea (3). A French study of 46 GFAP-IgG positive patients reported that 2 patients (4%) had intractable hiccups without apparent AP lesions on MRI (4). In our study, the incidence of APS was 9.3%, with all cases occurring in the early disease stage, consistent with the study by Deng et al. (11).

APS is an important phenomenon in neuroimmunology and demyelinating diseases (6). It is usually reported in NMOSD, especially in AQP4-IgG positive patients (23). Studies have shown a high prevalence of APS attacks in AQP4-IgG positive patients (onset: 7.1–14.9%; total: 17–43%) (23–28). APS was previously thought to be a way to distinguish NMOSD from A-GFAP-A. A study comparing GFAP-IgG and AQP4-IgG related myelitis found that 20% of AQP4-IgG related myelitis patients experienced INVH as prodromal symptoms, while GFAP-IgG related myelitis patients did not. This suggested that APS can help to differentiate these two disorders (29).

Isolated APS attacks are common in NMOSD but rare reported in A-GFAP-A (11, 23). None of our patients had isolated APS, as all experienced additional neurological events throughout their clinical course. Two patients developed other neurological symptoms more than 1 month after APS occurrence. However, isolated APS can occasionally be the only initial presentation of A-GFAP-A (11), making it challenging to differentiate from digestive disorders. Some patients were initially admitted to the gastroenterology department due to isolated INVH, resulting in misdiagnosis and delayed diagnosis.

Our study found that APS in A-GFAP-A differed from APS in NMOSD. The proportion of female patients in AQP4 + NMOSD was higher, consistent with the known female dominance in NMOSD. Additionally, hiccups were more common in A-GFAP-A than in AQP4 + NMOSD. We speculated that hiccups may draw clinicians’ attention to consider APS as a potential diagnosis. Nausea and vomiting can be a premonitory symptoms of A-GFAP-A, thus leading to the overlooking of APS. Notably, CSF inflammation in patients with A-GFAP-A was more severe than that in AQP4 + NMOSD, consistent with previous studies (29, 30).

MRI imaging of APS shows the lesion is located in the dorsal medulla oblongata including AP. This lesion can be isolated or contiguous with an upper cervical cord lesion. Similar to NMOSD, sagittal MRI images show longitudinally extensive linear or patchy cervical lesions to AP, known as linear medullary lesions or linear medullospinal lesions. Axial view reveals that medullary oblongata lesions involve the central canal or pericanal regions, extending into the fourth ventricle (11, 31). Previously, scholars believed that longitudinally extensive spinal cord lesions with a “linear sign” or an “inverted V” sign in medulla oblongata of patients with INVH were highly specific to NMOSD (24, 31). However, our study found that these signs were also observed in GFAP-IgG positive patients. Moreover, APS in A-GFAP-A also exhibited specific imaging findings, such as leptomeningeal enhancement and periventricular radial linear enhancement, which could provide valuable information for discrimination.

The diagnosis of APS emphasizes the importance of clinical manifestations rather than MRI features, as clinical symptoms do not always match imaging findings (5, 23, 31). When unexplained INVH persists for more than 48 h, the diagnosis of APS can be made even in the absence of MRI lesions in AP (31). In our study, 2 patients showed no obvious abnormalities in AP, which may be due to the timing of MRI examination. The lesion could have resolved by the time of imaging or may not yet be visible due to the early stage of the disease. Therefore, follow-up MRI should be considered if necessary. Additionally, a patient’s mild condition may result in a negative MRI (14, 15).

The AP, located on the floor of the fourth ventricle and in the dorsal portions of the medulla oblongata and nucleus tractus solitarius (23, 31), is considered the chemosensitive center for nausea and vomiting center. This region is highly vascularized and lacks the normal blood–brain barrier, facilitating the exchange of proteins and peptides between blood circulation and CSF (6). The pathogenesis of APS is irritant lesions involving the AP and adjacent structures such as nucleus tractus solitarius and nucleus ambiguous, leading to clinical symptoms like nausea and vomiting. AP highly expresses AQP4, making it susceptible to attack by AQP4-IgG. Similarly, GFAP-positive astrocytes are present within the AP which is surrounded by a dense zone of highly GFAP-reactive astrocytes. GFAP autoimmunity may target this region, contributing to APS (32).

GFAP-IgG can coexist with other neuronal antibodies, and AQP4-IgG is a common coexisting antibody (1, 2). In our study, 2 patients were positive for AQP4-IgG and exhibited typical MRI features of APS in NMOSD, such as the “linear sign” and “inverted V sign.” These patients met both the characteristics of A-GFAP-A and the diagnostic criteria for NMOSD. Therefore, it is a clinical challenge to make a suitable diagnosis and classification when overlapping antibodies occur simultaneously. A previous study found there was no critical difference between A-GFAP-A with and without overlapping syndrome. The author believed that mixed phenotypes indicated the coexistence of two simultaneously active immune mechanisms (33). However, unlike AQP4, GFAP is an intracellular antigen that is probably not directly pathogenic but a biomarker of cytotoxic T-cell-mediated autoimmune response (1–3). Especially in patients with overlapping syndrome, pathogenic antibodies such as AQP4-IgG further reinforce the bystander role of GFAP-IgG. AQP4-IgG may initiate a primary inflammatory event and disrupt astrocytic function, while GFAP autoimmunity occurs as a secondary phenomenon. The pathology of A-GFAP-A is heterogeneous (34). Autopsy findings of a patient with autoimmune meningoencephalomyelitis associated with GFAP antibody were nonspecific and did not demonstrate astrocyte involvement (35). These pathology, as well as coexisting antibodies and non-pathogenic GFAP-IgG, contribute to the heterogeneity of symptoms. Therefore, the clinical spectrum of A-GFAP-A might be broader.

APS in A-GFAP-A should be distinguished from the following situations: (1) A-GFAP-A may initially present with nonspecific flu-like prodromal symptoms, such as nausea and vomiting, that can mimic the APS (3, 29). (2) This disease can lead to hyponatremia, causing gastrointestinal dysfunction. (3) A-GFAP-A patients may exhibit autonomic dysfunction. GFAP autoimmunity directly targets enteric glial cells, which are similar to astrocytes and express GFAP, leading to gastroparesis and hiccups (1, 14, 36). APS is reversible. INVH can improve spontaneously without immunotherapy, with lesions disappearing on MRI (11). However, disabling encephalomyelitis occurring simultaneously with or following an APS attack may lead to a poor prognosis.

Our study has several limitations. First, it is a retrospective study with a limited number of patients, which restricted the collection of detail clinical characteristics, such as the severity and frequency of symptom, and titers of GFAP-IgG. Second, we did not conduct a comprehensive gastroenterological evaluation of all patients. Third, some patients did not receive radiographic follow-up due to the rapid improvement of symptoms. Additionally, comprehensive antibody testing was not conducted throughout the disease course. Thus, it is unknown other pathogenic neuronal antibodies were present. Moreover, for the comparison of APS in A-GFAP-A and AQP4 + NMOSD, due to the retrospective and exploratory nature of the study, the *p* values should not be viewed as confirmatory.

In conclusion, APS is a relatively rare manifestation of A-GFAP-A and often presents as an initial symptom, potentially leading to misdiagnosis. Therefore, recognizing accompanying symptoms and conducting GFAP-IgG detection are valuable for accurate diagnosis. Early diagnosis and prompt immunotherapy are essential to prevent severe neurologic deficits. We suggest that A-GFAP-A may be an etiology of APS, and patients presenting with APS-like symptoms should undergo comprehensive antibody testing. Further research is necessary to enrich the spectrum of A-GFAP-A.

## Data Availability

The original contributions presented in the study are included in the article/supplementary material, further inquiries can be directed to the corresponding author.

## References

[1] Fang B McKeon A Hinson SR Kryzer TJ Pittock SJ Aksamit AJ. Autoimmune glial fibrillary acidic protein astrocypathy: a novel meningoencephalomyelitis. JAMA Neurol. (2016) 73:1297–307. doi: 10.1001/jamaneurol.2016.2549, PMID: 27618707

[2] Flanagan EP Hinson SR Lennon VA Fang B Aksamit AJ Morris PP. Glial fibrillary acidic protein immunoglobulin G as biomarker of autoimmune astrocypathy: analysis of 102 patients. Ann Neurol. (2017) 81:298–309. doi: 10.1002/ana.24881, PMID: 28120349

[3] Long Y Liang J Xu H Huang J Yang J Gao C. Autoimmune glial fibrillary acidic protein astrocytopathy in Chinese patients: a retrospective study. Eur J Neuol. (2018) 25:477–83. doi: 10.1111/ene.13531, PMID: 29193473

[4] Gravier-Dumonceau A Ameli R Rogemond V Ruiz A Joubert B Muñiz-Castrillo S. Glial fibrillary acidic protein astrocytopathy: a French cohort study. Neurology. (2022) 98:e653–68. doi: 10.1212/WNL.0000000000013087, PMID: 34799461 PMC8829963

[5] Wingerchuk DM Banwell B Bennett JL Cabre P Carroll W Chitnis T. International consensus diagnostic criteria for neuromyelitis optica spectrum disorders. Neurology. (2015) 85:177–89. doi: 10.1212/WNL.0000000000001729, PMID: 26092914 PMC4515040

[6] Camara-Lemarroy CR Burton JM. Area postrema syndrome: a short history of a pearl in demyelinating diseases. Mult Scler. (2019) 25:325–9. doi: 10.1177/1352458518813105, PMID: 30463481

[7] Zeiner PS Brandhofe A Müller-Eschner M Steinmetz H Pfeilschifter W. Area postrema syndrome as frequent feature of Bickerstaff brainstem encephalitis. Ann Clin Transl Neurol. (2018) 5:1534–42. doi: 10.1002/acn3.666, PMID: 30564620 PMC6292382

[8] Tanaka Y Koga Y Takada H. Pilocytic astrocytoma at the medulla oblongata dorsal surface presenting as intractable hiccups. Pediatr Neurol. (2015) 52:254–5. doi: 10.1016/j.pediatrneurol.2014.10.029, PMID: 25443583

[9] Lavezzi AM Cappiello A Termopoli V Bonoldi E Matturri L. Sudden infant death with area postrema lesion likely due to wrong use of insecticide. Pediatrics. (2015) 136:e1039–42. doi: 10.1542/peds.2015-0425, PMID: 26371202

[10] Ciron J Sourdrille F Biotti D Tchoumi T Ruiz A Bernard-Valnet R. Area postrema syndrome: another feature of anti-GFAP encephalomyelitis. Mult Scler. (2020) 26:253–5. doi: 10.1177/1352458518817992, PMID: 30663514

[11] Deng B Wang J Yu H Jin L Qiu Y Liu X. Area postrema syndrome in autoimmune glial fibrillary acidic protein astrocytopathy: a case series and literature review. Neurol Neuroimmunol Neuroinflamm. (2022) 9:e200029. doi: 10.1212/NXI.0000000000200029, PMID: 36163176 PMC9513980

[12] Liang X Shen Y. Area postrema syndrome with linear enhancement along the surface of the brainstem and fourth ventricle in autoimmune GFAP astrocytopathy. BMC Neurol. (2023) 23:78. doi: 10.1186/s12883-023-03126-5, PMID: 36805663 PMC9940409

[13] Gao X Tang Y Yang G Wei W. Autoimmune glial fibrillary acidic protein astrocytopathy associated with area postrema syndrome: a case report. Front Neurol. (2021) 12:803116. doi: 10.3389/fneur.2021.803116, PMID: 35002942 PMC8739890

[14] Iwami K Nomura T Seo S Nojima S Tsuzaka K Kimura A. Autoimmune glial fibrillary acidic protein astrocytopathy presenting with area postrema syndrome-like symptoms without medulla oblongata lesions. Neuroimmunomodulation. (2022) 29:433–8. doi: 10.1159/000524344, PMID: 35421859

[15] Dang J Lei S Chen J. Autoimmune glial fibrillary acidic protein astrocytopathy presented as isolated area postrema syndrome: a case report. BMC Neurol. (2022) 22:271. doi: 10.1186/s12883-022-02802-2, PMID: 35858856 PMC9297591

[16] Zhang Y Bhekharee AK Zhang X. NMOSD or GFAP astrocytopathy? A case report. Mult Scler Relat Disord. (2020) 43:102202. doi: 10.1016/j.msard.2020.102202 32474284

[17] Adachi H Shiomi Y Kimura A Shimohata T Yoneda Y Kageyama Y. A case of autoimmune glial fibrillary acidic protein (GFAP) astrocytopathy. Rinsho Shinkeigaku. (2021) 61:401–4. doi: 10.5692/clinicalneurol.cn-001575, PMID: 34011813

[18] Ma W Huang C Yang L Luo J. MRI findings of autoimmune glial fibrillary acidic protein astrocytopathy involving infratentorial: case report. Radiol Case Rep. (2022) 17:2515–8. doi: 10.1016/j.radcr.2022.04.032, PMID: 35601378 PMC9114157

[19] Koh PX Tay KY Yeo T Singh DR Koh JS Thirugnanam UN. Glial fibrillary acidic protein astrocytopathy in a patient with recent mRNA SARS-CoV-2 vaccination. Neuroimmunol Rep. (2022) 2:100053. doi: 10.1016/j.nerep.2021.100053

[20] Patel N Rao VA Heilman-Espinoza ER Lai R Quesada RA Flint AC. Simple and reliable determination of the modified Rankin scale score in neurosurgical and neurological patients: the mRS-9Q. Neurosurgery. (2012) 71:971–5. doi: 10.1227/NEU.0b013e31826a8a56, PMID: 22843133

[21] Hol EM Pekny M. Glial fibrillary acidic protein (GFAP) and the astrocyte intermediate filament system in diseases of the central nervous system. Curr Opin Cell Biol. (2015) 32:121–30. doi: 10.1016/j.ceb.2015.02.004, PMID: 25726916

[22] Yang Z Wang KK. Glial fibrillary acidic protein: from intermediate filament assembly and gliosis to neurobiomarker. Trends Neurosci. (2015) 38:364–74. doi: 10.1016/j.tins.2015.04.003, PMID: 25975510 PMC4559283

[23] Shosha E Dubey D Palace J Nakashima I Jacob A Fujihara K. Area postrema syndrome: frequency, criteria, and severity in AQP4-IgG-positive NMOSD. Neurology. (2018) 91:e1642–51. doi: 10.1212/WNL.0000000000006392, PMID: 30258024 PMC6205685

[24] Zhou C Liao L Sun R Wang J di W Zhu Y. Area postrema syndrome as initial manifestation in neuromyelitis optica spectrum disorder patients: a retrospective study. Rev Neurol. (2021) 177:400–6. doi: 10.1016/j.neurol.2020.07.019, PMID: 33081997

[25] Iorio R Lucchinetti CF Lennon VA Farrugia G Pasricha P Weinshenker BG. Intractable nausea and vomiting from autoantibodies against a brain water channel. Clin Gastroenterol Hepatol. (2013) 11:240–5. doi: 10.1016/j.cgh.2012.11.021, PMID: 23211959 PMC3581743

[26] Takahashi T Miyazawa I Misu T Takano R Nakashima I Fujihara K. Intractable hiccup and nausea in neuromyelitis optica with anti-aquaporin-4 antibody: a herald of acute exacerbations. J Neurol Neurosurg Psychiatry. (2008) 79:1075–8. doi: 10.1136/jnnp.2008.145391, PMID: 18420727

[27] Pittock SJ Weinshenker BG Lucchinetti CF Wingerchuk DM Corboy JR Lennon VA. Neuromyelitis optica brain lesions localized at sites of high aquaporin 4 expression. Arch Neurol. (2006) 63:964–8. doi: 10.1001/archneur.63.7.964, PMID: 16831965

[28] Kremer L Mealy M Jacob A Nakashima I Cabre P Bigi S. Brainstem manifestations in neuromyelitis optica: a multicenter study of 258 patients. Mult Scler. (2014) 20:843–7. doi: 10.1177/1352458513507822, PMID: 24099751

[29] Sechi E Morris PP McKeon A Pittock SJ Hinson SR Weinshenker BG. Glial fibrillary acidic protein IgG related myelitis: characterisation and comparison with aquaporin-4-IgG myelitis. J Neurol Neurosurg Psychiatry. (2019) 90:488–90. doi: 10.1136/jnnp-2018-318004, PMID: 30032117

[30] Xiao J Zhang S Chen X Tang Y Chen M Shang K. Comparison of clinical and radiological characteristics in autoimmune GFAP astrocytopathy, MOGAD and AQP4-IgG^+^ NMOSD mimicking intracranial infection as the initial manifestation. Mult Scler Relat Disord. (2022) 66:104057. doi: 10.1016/j.msard.2022.104057 35870369

[31] Dubey D Pittock SJ Krecke KN Flanagan EP. Association of extension of cervical cord lesion and area postrema syndrome with neuromyelitis optica spectrum disorder. JAMA Neurol. (2017) 74:359–61. doi: 10.1001/jamaneurol.2016.5441, PMID: 28097302

[32] Willis CL Garwood CJ Ray DE. A size selective vascular barrier in the rat area postrema formed by perivascular macrophages and the extracellular matrix. Neuroscience. (2007) 150:498–509. doi: 10.1016/j.neuroscience.2007.09.023, PMID: 17945430

[33] Yang X Xu H Ding M Huang Q Chen B Yang H. Overlapping autoimmune syndromes in patients with glial fibrillary acidic protein antibodies. Front Neurol. (2018) 9:251. doi: 10.3389/fneur.2018.00251, PMID: 29755396 PMC5932346

[34] Shan F Long Y Qiu W. Autoimmune glial fibrillary acidic protein astrocytopathy: a review of the literature. Front Immunol. (2018) 9:2802. doi: 10.3389/fimmu.2018.02802, PMID: 30568655 PMC6290896

[35] Yamakawa M Hogan KO Leever J Jassam YN. Autopsy case of meningoencephalomyelitis associated with glial fibrillary acidic protein antibody. Neurol Neuroimmunol Neuroinflamm. (2021) 8:e1081. doi: 10.1212/NXI.0000000000001081, PMID: 34642236 PMC8515200

[36] Mekeon A Benarroch EE. Glial fibrillary acid protein: functions and involvement in disease. Neurology. (2018) 90:925–30. doi: 10.1212/WNL.0000000000005534 29653988

